# Construction and validation of a prognostic nomogram for predicting the survival of HIV/AIDS adults who received antiretroviral therapy: a cohort between 2003 and 2019 in Nanjing

**DOI:** 10.1186/s12889-021-12249-8

**Published:** 2022-01-06

**Authors:** Fangfang Jiang, Yuanyuan Xu, Li Liu, Kai Wang, Lu Wang, Gengfeng Fu, Liping Wang, Zhongjie Li, Junjie Xu, Hui Xing, Ning Wang, Zhengping Zhu, Zhihang Peng

**Affiliations:** 1 Department of Epidemiology and Biostatistics, School of Public Health, Nanjing Medical University, Nanjing, 211166 Jiangsu China; 2 Department of AIDS and STDs control and prevention, Nanjing Municipal Center for Disease Control and Prevention, Nanjing, 210003 Jiangsu China; 3 Jiangsu Provincial Key Laboratory of Geriatrics, Department of Geriatrics, The First Affiliated Hospital of Nanjing Medical University, Nanjing, Jiangsu China; 4 National Center for AIDS/STD Control and Prevention, Chinese Center for Disease Control and Prevention, Beijing, 102206 China; 5 Department of STDs/AIDS Prevention and Control, Jiangsu Center for Disease Prevention and Control, Jiangsu, 210027 China; 6 Division of Infectious Diseases, Key Laboratory of Surveillance and Early-warning on Infectious Disease, Chinese Center for Disease Control and Prevention, Beijing, 102206 China; 7 Department of Epidemiology and Biostatistics, School of Public Health, China Medical University, Beijing, 110001 China

**Keywords:** HIV/AIDS, Antiretroviral therapy, Prognostic model, Nomogram

## Abstract

**Background:**

Great achievements have been achieved by free antiretroviral therapy (ART). A rapid and accurate prediction of survival in people living with HIV/AIDS (PLHIV) is needed for effective management. We aimed to establish an effective prognostic model to forecast the survival of PLHIV after ART.

**Methods:**

The participants were enrolled from a follow-up cohort over 2003-2019 in Nanjing AIDS Prevention and Control Information System. A nested case-control study was employed with HIV-related death, and a propensity-score matching (PSM) approach was applied in a ratio of 1:4 to allocate the patients. Univariable and multivariable Cox proportional hazards analyses were performed based on the training set to determine the risk factors. The discrimination was qualified using the area under the curve (AUC) and concordance index (C-Index). The nomogram was calibrated using the calibration curve. The clinical benefit of prognostic nomogram was assessed by decision curve analysis (DCA).

**Results:**

Predictive factors including CD4 cell count (CD4), body mass index (BMI) and hemoglobin (HB) were determined and incorporated into the nomogram. In the training set, AUC and C-index (95% CI) were 0.831 and 0.798 (0.758, 0.839), respectively. The validation set revealed a good discrimination with an AUC of 0.802 and a C-index (95% CI) of 0.786 (0.681, 0.892). The calibration curve also exhibited a high consistency in the predictive power (especially in the first 3 years after ART initiation) of the nomogram. Moreover, DCA demonstrated that the nomogram was clinically beneficial.

**Conclusion:**

The nomogram is effective and accurate in forecasting the survival of PLHIV, and beneficial for medical workers in health administration.

**Supplementary Information:**

The online version contains supplementary material available at 10.1186/s12889-021-12249-8.

## Introduction

Over the past 30 years, HIV has become a major global public health challenge [1]. In China, free antiretroviral therapy (ART), launched in 2003, has proven to efficiently recover CD4 cell count (CD4), lower viral load (VL) and curb HIV transmission [2]. Nevertheless, the poor prognosis of people living with HIV/AIDS (PLHIV) after ART remains a concern [3, 4]. It is essential to create a tool to rapidly and accurately predict death risk among PLHIV.

Studies have shown that CD4, CD8 cell count (CD8), and VL before treatment are closely associated with the mortality of PLHIV [5–13]. Clinical indicators are reported to have a close association with death risk of PLHIV [5, 7–16]. Some laboratory indicators, such as hemoglobin (HB), platelet-related indexes, are also related to the progression and mortality of HIV-related diseases after ART [3, 17–23].

Since the combination of several independent indicators, rather than a single predictive factor, has a stronger predictive power, several scoring systems based on the multiple risk factors have been proposed to forecast the mortality of PLHIV. However, there still lacks a widely-held effective scoring system to predict the survival of ART-treated PLHIV.

In recent years, various multi-factor models have been designed to estimate disease outcomes. A risk-scoring system can be established according to the recommendation of Transparent Reporting of a multivariable prediction model for Individual Prognosis or Diagnosis (TRIPOD) [24]. Nomogram is convenient to predict the prognosis of patients [25]. Previous nomograms have failed to assess the outcomes of ART. For example, in the model by Margaret et al. [26], a concordance index (C-Index) of 0.75 (95% CI: 0.74-0.81) in the training set and a C-Index of 0.69 (95% CI: 0.59-0.77) in the validation set were presented. This model achieves a satisfactory performance, but far from excellent. Few prognostic models based on PLHIV after receiving ART have presented good discrimination and calibration. In the model established by Hou et al. [27], the C-Indexes are 0.91 (95% CI: 0.86-0.97) in the training set and 0.92 (95% CI: 0.82-1.00) in the validation set.

In the present study, to build a simple and effective prognostic model to forecast the survival of PLHIV after ART, a nested case-control study was employed with HIV-related mortality events, and a propensity-score matching (PSM) approach was applied to allocate the patients in a ratio of 1:4. To make the model more reliable and robust, bootstrap was used for internal validation. The discrimination and calibration of the model were evaluated based on the training set and validation set. Decision curve analysis (DCA) was also used to evaluate the performance of the nomogram.

## Materials and methods

### Study design

**Fig. 1:**
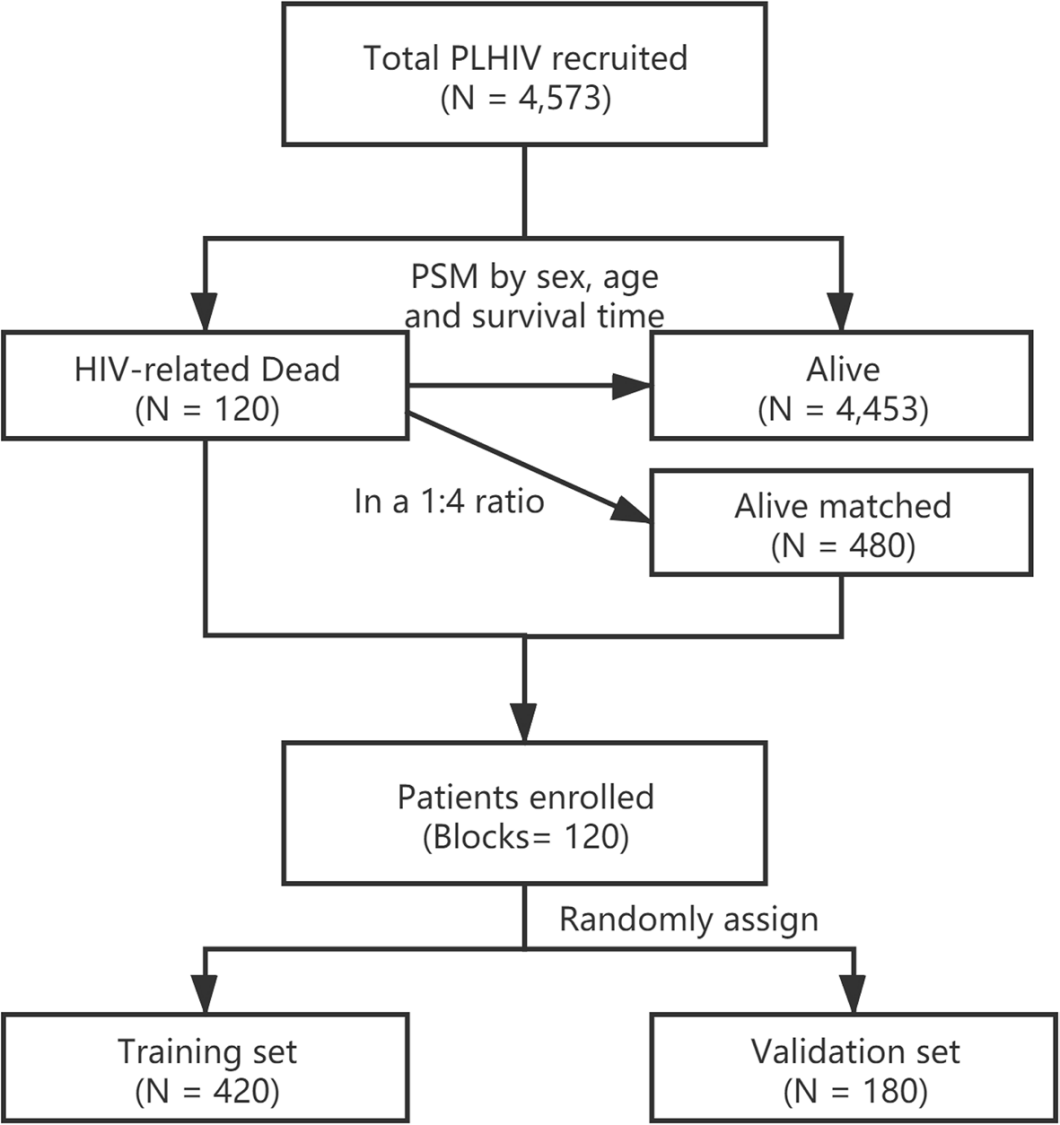
A flowchart of predicted HIV-related survival of people living with HIV/AIDS (PLHIV) using nomogram model

The data used in this study were extracted from patients who received ART between 2003 and 2019 from Nanjing AIDS Prevention and Control Information System (AIDS-PCIS). All patients received a free ART containing at least three antiviral medicines. The follow-up started after ART initiation and the participants were visited every 3 months. The observation end point was December 31, 2019, and the outcome was death. The survival time was defined as the duration from ART initiation to death or December 31, 2019. The inclusion criteria included: (1) living in Nanjing; (2) being visited at least once; (3) being over 18 years old when ART started; and (4) having complete laboratory test data before starting ART. At the end of the follow-up, a total of 4573 patients met the inclusion criteria. Among them, 120 patients died of HIV/AIDS-related diseases and were determined as the cases in the nested case-control study. The flowchart of recruitment participants is shown in the Fig. 1.

### Data collection

Demographic data and clinical information were retrieved from face-to-face surveys at the patients’ enrollment or extracted from their medical records using a structured questionnaire designed specifically for AIDS-PCIS. The information included the date of birth, gender, height, weight, marital status, infection route and WHO clinical stage. The age of the patient was calculated from the date of birth to the date of starting ART. Body mass index (BMI) was calculated using the following formula: BMI = weight (kg) / (height (m) × height (m)).

The laboratory testing data were obtained from the Nanjing Center for Disease Control and Prevention (CDC) or local hospitals. The laboratory testing indicators included CD4, white blood cell (WBC), blood platelets (PLT), HB, serum creatinine (CR), triglycerides (TG), total cholesterol (TC), fasting blood glucose (FBG), aspartate aminotransferase (AST), alanine aminotransferase (ALT) and total bilirubin (TBIL). All these laboratory tests were carried out by the trained technical personnel strictly following clinical guidelines at each visit in the central laboratory of local hospitals or Nanjing CDC.

Routine blood biochemical indexes, such as TG, TC, FBG, CR, AST, ALT, and TBIL, were measured using a Beckman AU5800 automatic biochemical analyzer (Beckman COULTER K., Japan). Other indexes including WBC, HB and PLT were evaluated by Sysmex Xe-2100 automatic blood cell analyzer (Sysmex Corporation, Japan). CD4 was determined by the BD FACSCalibur flow cytometer (Becton Dickinson Corporation, USA).

### Statistical analysis

#### Data processing

For a multi-factor regression model, there is no simple method to estimate its proper sample size. When the number of predictors is much larger than that of outcomes, overfitting may occur. Previous literature showed that in the conservative estimation, one prediction factor requires at least 10 effective outcomes. In this study, there were 120 cases with effective outcomes, so the number of predictors should be less than 12.

**Fig. 2:**
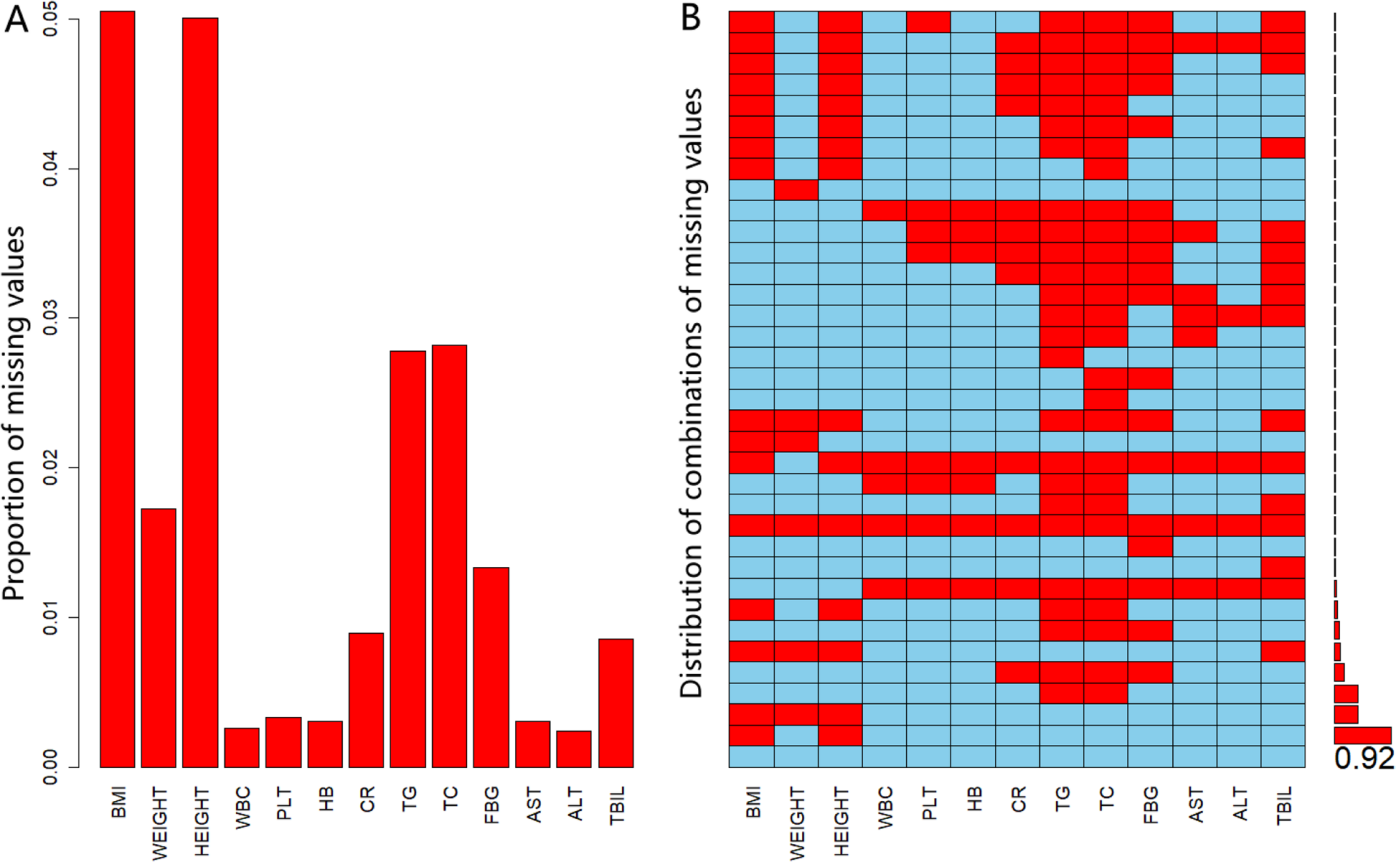
Proportion of missing values (**A**) and distribution of combinations of missing values (**B**) in training set. Abbreviations: BMI = body mass index; WBC = white blood cell; PLT = blood platelet; HB = hemoglobin; CR = creatinine; TG = triglyceride; TC = total cholesterol; FBG = fasting blood glucose; AST = aspartate aminotransferase; ALT = alanine aminotransferase; TBIL = total bilirubin

Since directly dropping the data with missing values might lead to selection bias, or decrease the power of a test, missing value imputation was applied to obtain suitable values by employing the values of other variables before data analysis. The results were listed in Fig. 2. A sensitivity analysis was carried out to evaluate the filling effect of the missing values (Table 1).

**Table 1:** Sensitivity analysis in imputation for missing data

Variables	Before imputation(mean ± standard deviation)	After imputation(mean ± standard deviation)	***P*** value
Weight, kg	64.8 ± 10.2	64.8 ± 10.1	0.953
Height, cm	172.0 ± 6.1	172.0 ± 6.1	0.828
BMI, kg/m2	21.9 ± 3.0	21.8 ± 3.0	0.564
WBC, 10^9^/L	5.7 ± 2.0	5.7 ± 2.0	0.991
PLT, 10^9^/L	188.7 ± 62.5	188.6 ± 62.5	0.961
HB, g/L	142.7 ± 30.9	142.7 ± 30.9	0.995
CR, mmol/L	71.3 ± 19.4	71.3 ± 19.4	0.932
TG, mmol/L	1.7 ± 2.3	1.7 ± 2.3	0.928
TC, mmol/L	4.3 ± 1.3	4.3 ± 1.3	0.976
FBG, mmol/L	5.6 ± 1.3	5.6 ± 1.3	0.922
AST, U/L	27.0 ± 27.2	27.1 ± 27.2	0.955
ALT, U/L	30.2 ± 29.2	30.2 ± 29.2	0.986
TBIL, mmol/L	12.0 ± 6.2	12.0 ± 6.2	0.964

Abbreviations: *BMI* body mass index, *WBC* white blood cell, *PLT* blood platelet, *HB* hemoglobin, *CR* creatinine, *TG* triglyceride, *TC* total cholesterol, *FBG* fasting blood glucose, *AST* aspartate aminotransferase, *ALT* alanine aminotransferase, *TBIL* total bilirubin

A total of 120 deaths caused by HIV/AIDS-related diseases were determined as the cases in the nested case-control study. S(60) was set as the index date (month). To ensure that all the subjects in the case group could have a matching control, PSM was applied in a ratio of 1:4 to determine the participants (a case was well matched with 4 controls in age, gender and index date) [28]. Finally, 600 subjects were included in this study with 120 dead and 480 alive PLHIV who were separated into 120 blocks.

#### Establishment and validation of prediction model

The patients were randomly split into a training set and a validation set in a ratio of 7:3. The comparability of the training set and validation set was then evaluated. Continuous variables with normal distribution were presented as mean ± standard deviation, and t-tests were used to infer the differences between the training and validation sets. The continuous variables with skewed distribution were described using median (first quartile, second quartile). The Wilcoxon rank-sum tests were employed for comparisons. Frequency (ratio) was utilized to describe the characteristics of categorical variables, and comparisons between the two sets were performed using chi-square tests or Fisher’s exact tests.

Then the data in the training set were used to fit a model and the data in validation set were applied to evaluate the efficacy of the model. Based on the data in the training set, univariable Cox proportional hazards analysis was performed for each variable. *P*-values of the variables were calculated based on the univariable Cox proportional hazards regression model. The variables with *p*-values less than or equal to 0.2 were included in a multivariable Cox proportional hazards regression model. After the multivariable analysis, the factors with *p*-value less than or equal to 0.05 were included in the prediction model. According to Occam’s Razor, the model with the fewest variables is the best [29]. Finally, we considered both the statistically significant risk factors and professionally significant factors, such as the difficulty of index measurement, the cost of measurement and the difficulty of application, and then determined the predictive factors and select a prediction model with the best predictive performance.

The repeatability and extrapolation of the prediction model should be evaluated. A strict evaluation of the prediction model should include internal validation and external validation. The internal validation is performed using the same dataset as the training set. This study employed the bootstrap resampling [30] for internal validation because of the lack of additional data to verify the model. The 1000 resampling performances of the model were averaged as the internal validation performance.

Discrimination and calibration are the two most common evaluation indicators. The discrimination of the prediction model is quantified using the area under the curve (AUC) and C-Index. The C-Index value ranges from 0 to 1. The closer C-Index is to 1, the better the discrimination of the model is. A C-Index of 0.5 indicates that the model has no predictive ability. When C-Index is less than 0.5, the model prediction is contrary to the actual results. In general, a C-Index of 0.7 indicates a good prediction performance of the model. However, discrimination cannot reflect whether the estimate of absolute risk of prediction model is accurate or not because it is only based on risk scores or the ranking of prediction probabilities. Calibration is a more accurate indicator to qualify the prediction model. In this study, the calibration of the model was evaluated using the calibration curve. We sorted the predicted probabilities of all participants from the smallest to the largest, and divided the patients into ten equal parts. The average predicted probability of patients in each divided part was used as x-axis and the proportion of actual events as y-axis. Ideally, the calibration graph was a straight line with an intercept of 0 and a slope of 1. The predictive ability of the model was also evaluated using decision curve analysis (DCA).

Integrated discrimination improvement (IDI), net reclassification index or improvement (NRI) and other indicators that are used to compare models or evaluate the increase in predictive performance of individual predictors were not discussed in the present study.

#### Presentation of nomogram

The prediction model was visualized and presented by a nomogram. To calculate the score of each variable at each level, a scoring standard was developed based on the standard regression coefficients of all variables. Then using the scores of these factors, we calculated a total score to indicate the survival probability of each patient.

All data analyses and figures were made using R software version 4.1.0. All hypothesis tests were two-sided, with an α level of 0.05.

## Results

### Establishment of prediction model

In this PSM-based nested case-control study, the characteristics of the 600 PLHIV (420 from the training set and 180 from the validation set) revealed that both sets were similar in all variables (Table 2).

**Table 2:** Baseline demographics and clinical characteristics of patients in the training set and the validation set

Variable	Training set (*N* = 420)	Validation set (*N* = 180)	*P* value
**Continuous variables**			
Age, year	50.00 (40.00,57.00)	48.00 (37.75,61.00)	0.376
Interval from onset to diagnosis, day	52.00 (33.00,159.50)	46.50 (26.00,149.00)	0.107
ALT, U/L	21.65 (15.57,32.92)	22.15 (16.00,32.65)	0.629
AST, U/L	22.15 (17.90,30.33)	23.45 (18.95,31.23)	0.173
BMI, kg/m^2^	21.85 (19.84,23.94)	21.48 (19.24,23.68)	0.106
CD4, cells/μL	259.50 (111.50,384.50)	243.50 (101.00,377.25)	0.79
TC, mmol/L	4.15 (3.55,4.82)	4.12 (3.48,4.80)	0.559
CR, *μ*mol/L	69.00 (61.00,77.00)	69.00 (59.83,78.00)	0.778
FBG, mmol/L	5.60 (5.10,6.09)	5.37 (4.91,5.92)	0.051
HB, g/L	136.00 (119.75,151.00)	136.00 (117.75,152.25)	0.956
PLT, 10^9^/L	172.00 (137.00,213.25)	167.50 (135.75,219.00)	0.808
TBIL, *μ*mol/L	10.25 (7.80,14.00)	10.30 (6.80,13.40)	0.203
TG, mmol/L	1.38 (1.03,1.87)	1.32 (0.96,1.97)	0.458
WBC, 10^9^/L	5.20 (4.10,6.38)	5.04 (4.01,6.38)	0.475
**Discrete variables**			
Gender, n(%)			
Male	386 (91.90)	160 (88.89)	0.276
Female	34 (8.10)	20 (11.11)	
Hepatitis B Virus, n(%)			
Negative	390 (92.86)	170 (94.44)	0.593
Positive	30 (7.14)	10 (5.56)	
Hepatitis C Virus, n(%)			
Negative	413 (98.33)	178 (98.89)	0.731
Positive	7 (1.67)	2 (1.11)	
Marital status, n(%)			
Unmarried	68 (16.19)	42 (23.33)	0.05
Married	352 (83.81)	138 (76.67)	
Shingles, n(%)			
No	375 (89.29)	165 (91.67)	0.458
Yes	45 (10.71)	15 (8.33)	
Infection route, n(%)			
Homosexual transmission	240 (57.14)	98 (54.44)	0.77
Heterosexual transmission	122 (29.05)	54 (30.00)	
Other transmission	58 (13.81)	28 (15.56)	
Baseline TB, n(%)			
No	407 (96.90)	175 (97.22)	1.00
Yes	13 (3.10)	5 (2.78)	
WHO clinical stage, n(%)			
I, II	189 (45.00)	75 (41.67)	0.655
III	102 (24.29)	43 (23.89)	
IV	129 (30.71)	62 (34.44)	
Continuous or intermittent fever, n(%)			
No	387 (92.14)	167 (92.78)	0.868
Yes	33 (7.86)	13 (7.22)	
Continuous diarrhea (> one month), n(%)			
No	397 (94.52)	165 (91.67)	0.202
Yes	23 (5.48)	15 (8.33)	

Abbreviations: *ALT* alanine aminotransferase, *AST* aspartate aminotransferase, *BMI* body mass index, *CD4* CD4 cell count, *WBC* white blood cell, *PLT* blood platelet, *HB* hemoglobin, *CR* creatinine, *TG* triglyceride, *TC* total cholesterol, *FBG* fasting blood glucose, *TBIL* total bilirubin, *TB* Tuberculosis

**Fig. 3:**
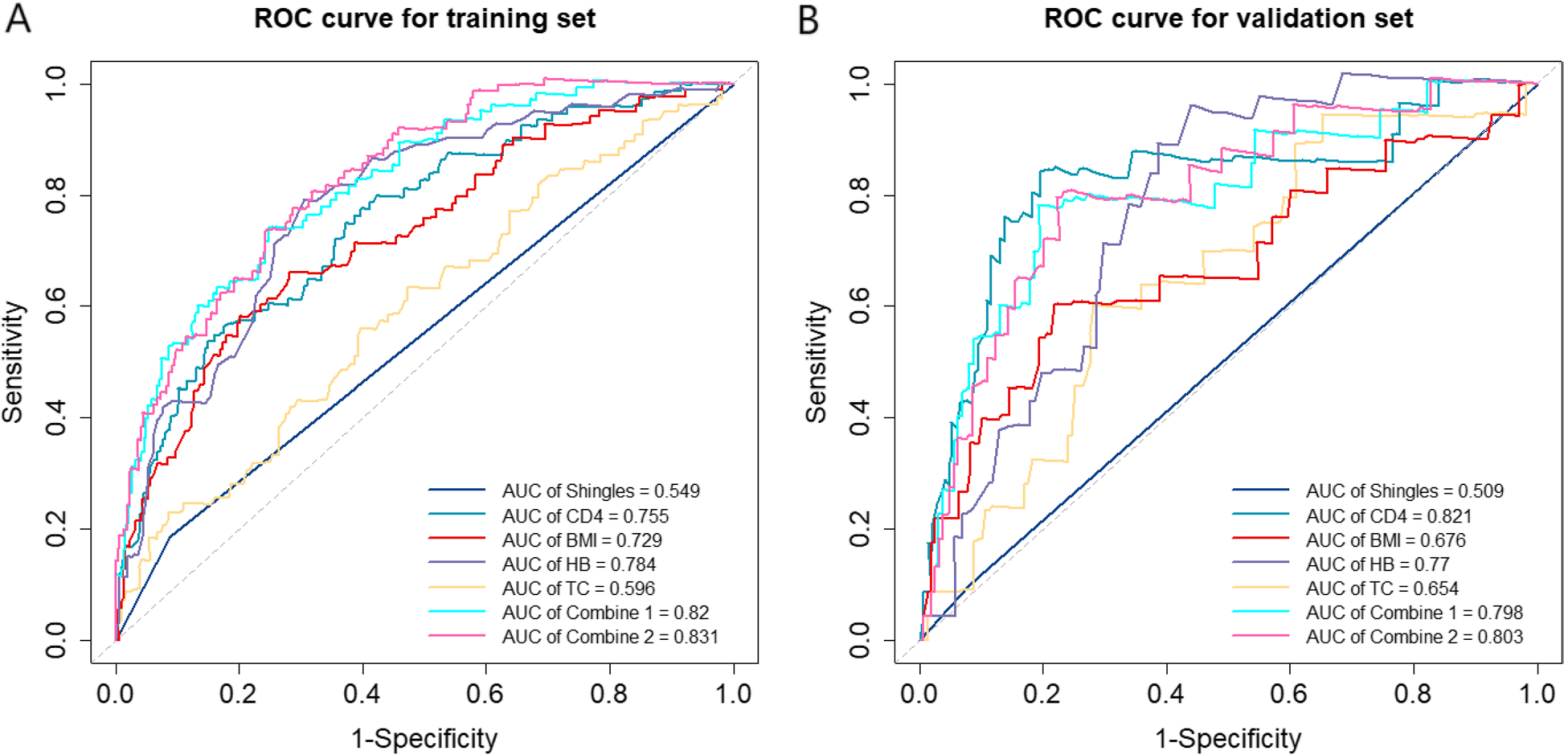
ROC curves of Shingles, CD4, BMI, HB and TC, combine 1 (Shingles, CD4, BMI, HB and TC) and combine 2 (CD4, BMI and HB) in the training set (**A**) and the validation set (**B**). Abbreviations: CD4 = CD4 cell count; BMI = body mass index; HB = hemoglobin; TC = total cholesterol

**Fig. 4:**
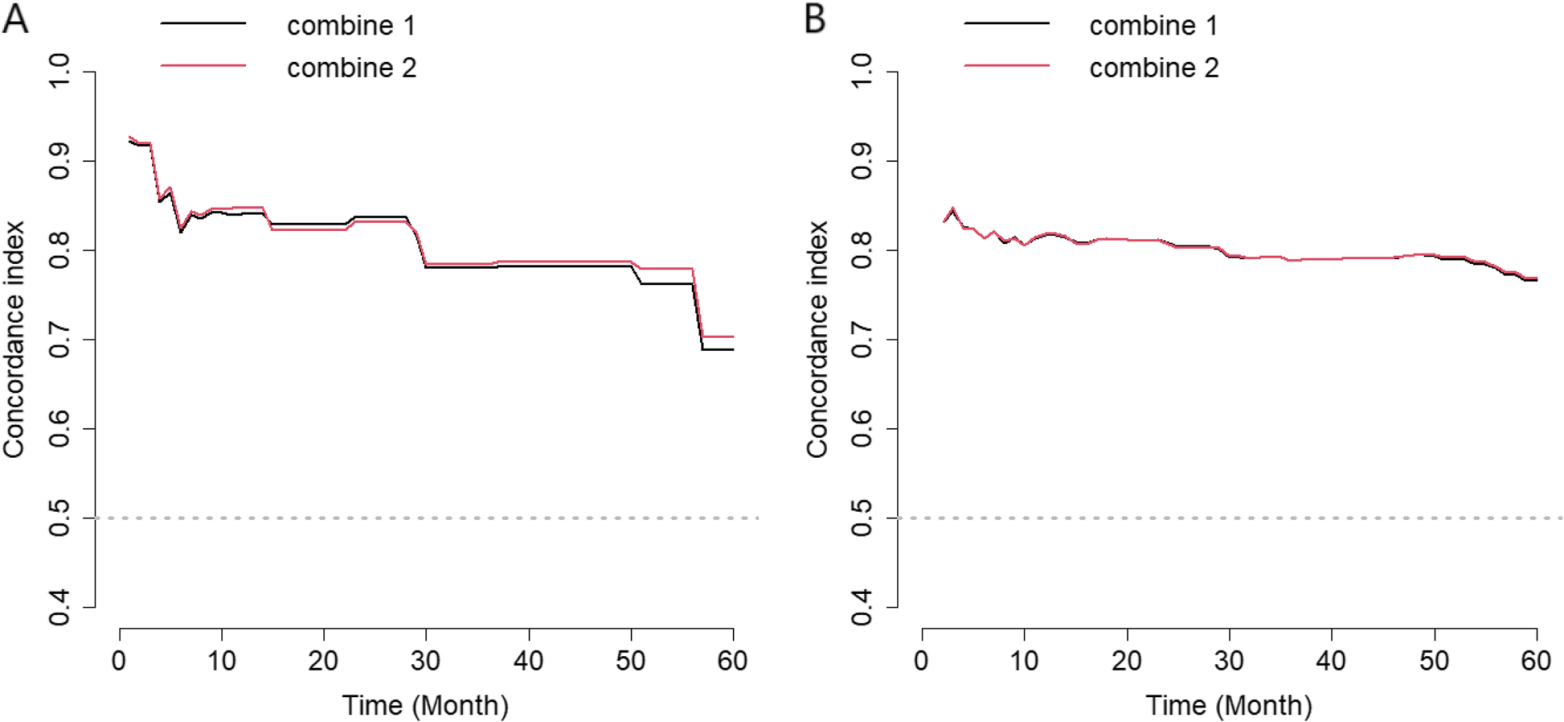
C-Indexes of combine 1 (Shingles, CD4, BMI, HB and TC) and combine 2 (CD4, BMI and HB) in the training set (**A**) and the validation set (**B**)

In the univariable Cox proportional hazards regression analysis of the training set, infection route, baseline Tuberculosis (TB), continuous diarrhea, continuous or intermittent fever, shingles, WHO clinical stage, CD4, BMI, HB, CR, TC, FBG, AST and ALT were detected to be statistically related to the mortality of PLHIV (Table 3). Variables with *p*-value less than or equal to 0.2 in the univariable analysis were included in the multivariable Cox proportional hazards regression model. To avoid multicollinearity caused by the strong relationship between WHO clinical stage and CD4, WHO clinical stage was not included in the multivariable Cox proportional hazards regression model. Shingles, CD4, BMI, HB and TC were found linked to HIV/AIDS-related death. In order to establish an optimal prediction model, the individual and combined performance of these factors were then evaluated using ROC analysis and C-Index. As shown in Fig. 3A, the AUCs of Shingles, CD4, BMI, HB and TC in the training set were 0.549, 0.755, 0.729, 0.669 and 0.596, respectively. The AUC of combine 1 (Shingles + CD4 + BMI + HB + TC) was 0.82, and the AUC of combine 2 (CD4 + BMI + HB) was 0.831. To compare the predictive performances of combine 1 and combine 2, their C-Indexes were calculated, and the results were 0.806 (95% CI: 0.766, 0.846) and 0.798 (95% CI: 0.758, 0.839), indicating both models had a prediction accuracy of around 80%. Besides, no statistically significant difference in the C-Indexes between combine model was observed (*P* = 0.957) (Fig. 4A). The discrimination between the two models was not large, but combine 2 involved fewer variables. Thus, combine 2 model was chosen and the three variables CD4, BMI and HB were preliminarily selected to construct a prediction model of three-year and five-year survival of PLHIV after ART.

**Table 3:** Univariable and multivariable Cox proportional hazards analysis of the training set

Variables	Univariable		Multivariable	
	HR (95%CI)	***P*** value	AHR (95%CI)	***P*** value
Female	1.364 (0.686,2.712)	0.376		
Marital Status (Married)	0.83 (0.496,1.388)	0.478		
Infection route				
Homosexual transmission	Ref	Ref	Ref	Ref
Heterosexual transmission	1.551 (0.984,2.446)	0.059#	1.327 (0.821, 2.145)	0.248
Other transmission	1.979 (1.142,3.428)	0.015*	1.193 (0.661, 2.154)	0.478
Baseline TB	2.024 (0.821,4.986)	0.125#	1.264 (0.491, 3.255)	0.584
HBV (Negative)	0.619 (0.227,1.689)	0.349		
HCV (Negative)	0.702 (0.098,5.04)	0.725		
Continuous diarrhea (> one month)	2.478 (1.286,4.775)	0.007*	2.271 (1.088, 4.742)	0.058
Continuous or intermittent fever	2.028 (1.141,3.605)	0.016*	0.905 (0.448, 1.680)	0.628
Shingles	1.905 (1.138,3.187)	0.014*	1.965 (1.140, 3.390)	0.018*
WHO clinical stage				
I, II	Ref	Ref	–	–
III	1.572 (0.908,2.721)	0.106#	–	–
IV	2.692 (1.678,4.318)	< .001***	–	–
Age, year	1.007 (0.99,1.023)	0.424		
Interval from onset to diagnosis, day	1.000 (1.000,1.000)	0.564		
CD4, cells/μL	0.995 (0.994,0.997)	< .001***	0.996 (0.995, 0.0.998)	< .001***
BMI, kg/m2	0.795 (0.74,0.853)	< .001***	0.875 (0.809, 0.946)	< .001***
WBC, 10^9^/L	0.969 (0.888,1.057)	0.476		
PLT, 10^9^/L	0.998 (0.995,1.001)	0.254		
HB, g/L	0.968 (0.961,0.975)	< .001***	0.975 (0.965, 0.984)	< .001***
CR, μmol/L	1.002 (1,1.005)	0.099#	1.002 (0.999, 1.005)	0.175
TG, mmol/L	0.963 (0.849,1.093)	0.562		
TC, mmol/L	0.707 (0.57,0.877)	0.002*	0.821 (0.670, 1.006)	0.038*
FBG, mmol/L	0.731 (0.59,0.905)	0.004*	0.938 (0.769, 1.145)	0.442
AST, U/L	1.011 (1.006,1.017)	< .001***	1.001 (0.989, 1.014)	0.951
ALT, U/L	1.008 (1.002,1.014)	0.008*	1.002 (0.992, 1.012)	0.647
TBIL, mmol/L	0.991 (0.957,1.027)	0.617		

Note: # *p* < 0.2; * *p* < 0.05; *** *p* < 0.001

Abbreviations: *CD4* CD4 cell count, *WBC* white blood cell, *PLT* blood platelet, *HB* hemoglobin, *CR* creatinine, *TG* triglyceride, *TC* total cholesterol, *FBG* fasting blood glucose, *AST* aspartate aminotransferase, *ALT* alanine aminotransferase, *TBIL* total bilirubin, *TB* Tuberculosis

### Validation of prediction model

To verify the efficacy of the model in predicting the survival of PLHIV, bootstrap resampling was used for internal validation of the model. In the validation set, the AUCs of Shingles, CD4, BMI, HB and TC were 0.509, 0.821, 0.676, 0.77 and 0.654 in the ROC analysis chart (Fig. 3B).

**Fig. 5:**
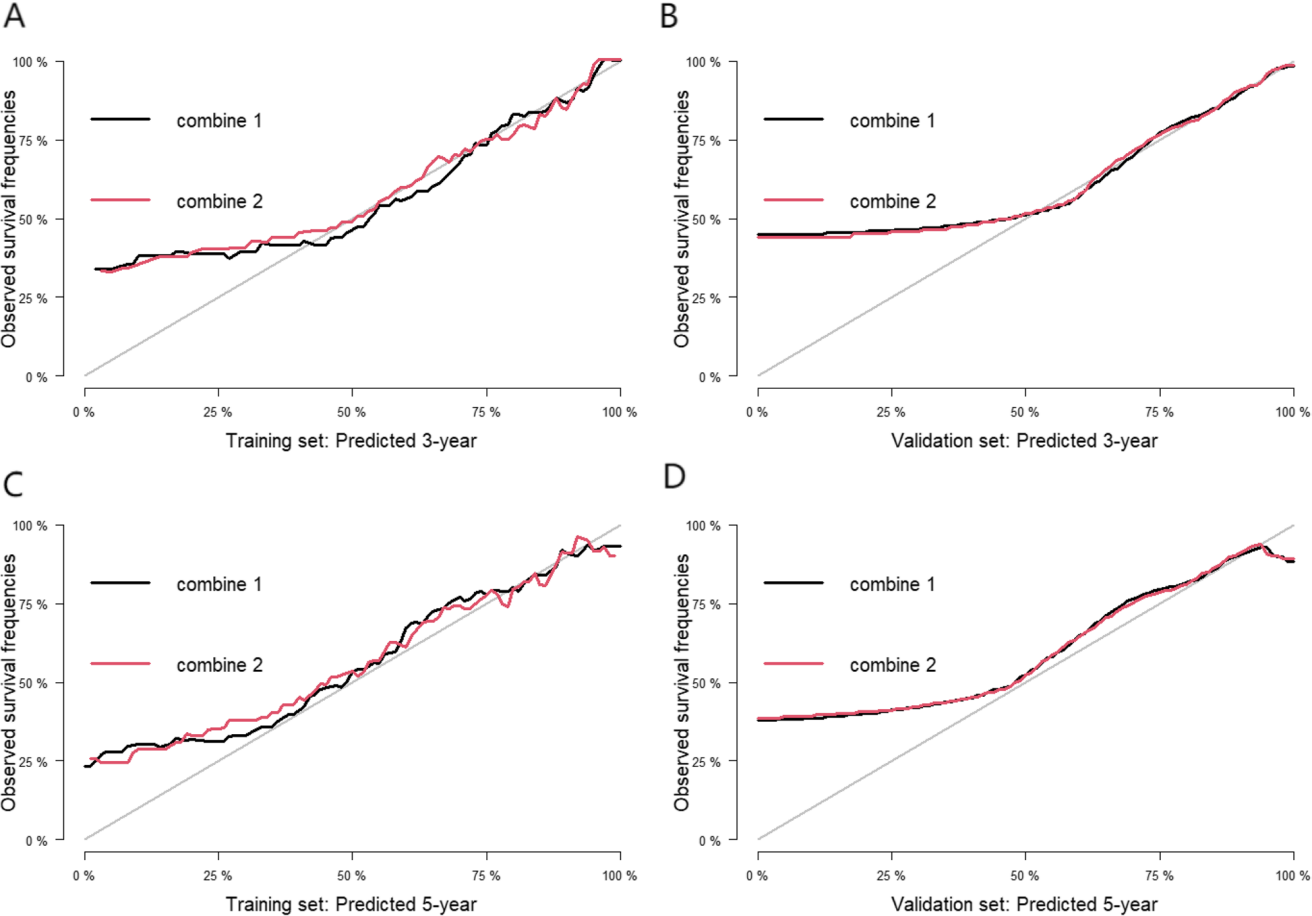
Calibration curves for predicting overall survival by combine 1 (Shingles, CD4, BMI, HB and TC) and combine 2 (CD4, BMI and HB) in the training set and the validation set. Notes: Calibration curves for 3-year overall survival (**A**), 5-year overall survival (**C**) in the training set; calibration curves for 3-year overall survival (**B**), 5-year overall survival (**D**) in the validation set

The AUC of combine 1 achieved 0.802, and the AUC of combine 2 (prediction model) was also 0.802. The C-Indexes of combine 1 (0.786; 95% CI: 0.679, 0.893) and combine 2 (0.786; 95% CI: 0.681, 0.892) were similar and the difference was not statistically significant (*P* = 0.998), which showed that the discrimination of combine 1 and combine 2 (prediction model) was not very large (Fig. 4B). The calibration curve also exhibited a high consistency in predicting the survival of PLHIV (especially in the first 3 years after ART initiation) (Fig. 5).

**Fig. 6:**
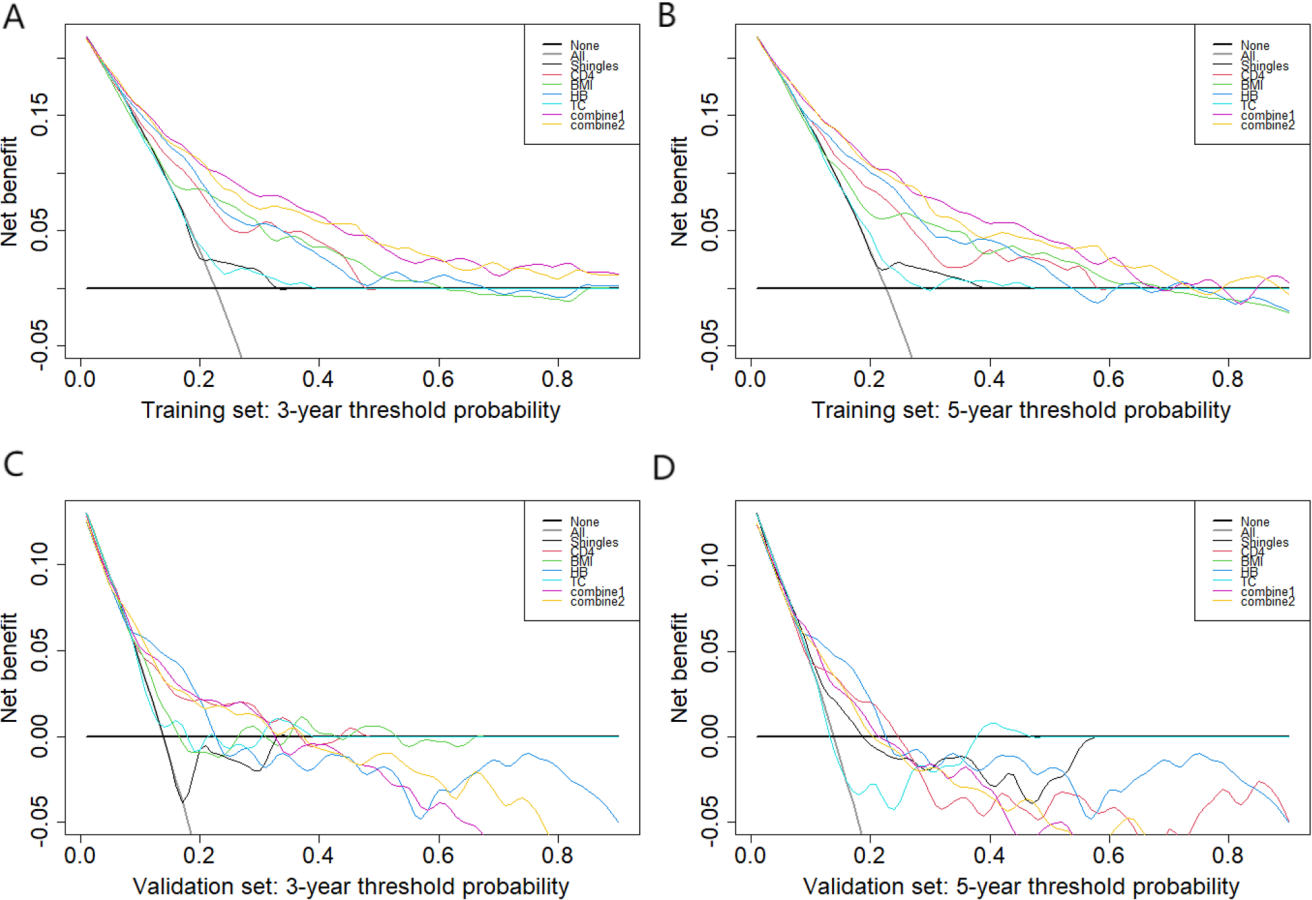
The DCA curve of Shingles, Diarrhea, WHO, CD4, BMI and HB, combine 1 (Shingles, CD4, BMI, HB and TC) and combine 2 (CD4, BMI and HB) in the training set and the validation set. Notes: DCA curve for 3-year overall survival (**A**), 5-year overall survival (**B**) in the training set; DCA curves for 3-year overall survival (**C**), 5-year overall survival (**D**) in the validation set. The horizontal axis represents the threshold probability, the probability of whether a patient receives treatment. The vertical axis represents the net benefit rate after the advantages minus the disadvantages. Under the same threshold probability, a larger net benefit implies that patients can obtain the maximum benefit using this model. The closer the curve in the DCA graph is to the top, the higher the value of the model diagnosis is. Abbreviations: CD4 = CD4 cell count; BMI = body mass index; HB = hemoglobin; TC = total cholesterol

As shown in Fig. 6, in both the training set and the validation set, the prediction model (combine 2) showed better performance. Overall, the DCA curve demonstrated that the prediction model (combine 2) could make valuable and profitable judgements. In addition, among the detected factors, CD4 was more beneficial than the other routine clinical laboratory indicators in predicting the three-year and five-year survival probabilities of PLHIV. In Fig. 6D, DCA curve showed that the prediction model had no good benefits in predicting five-year survival probabilities of PLHIV in the validation set.

### Performance of nomogram

**Fig. 7:**
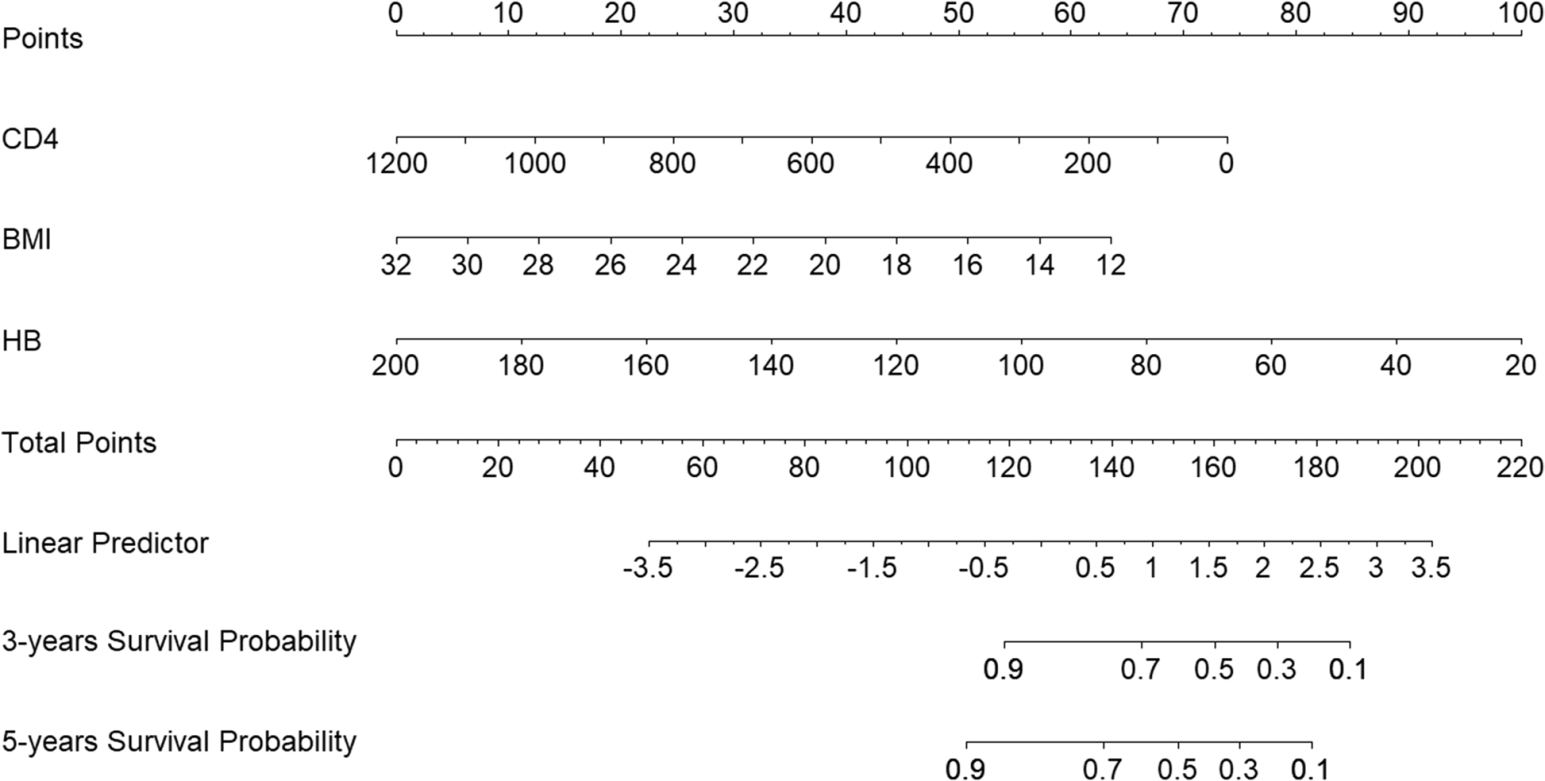
Nomogram of indexes for predicting HIV/AIDS-related survival of PLHIV after ART initiation. Abbreviations: CD4 = CD4 cell count; BMI = body mass index; HB = hemoglobin

A nomogram was drawn according to the determined prediction model. As seen in Fig. 7, each selected predictor was assigned with a score according to its value in the nomogram based on the established prediction model. Then a vertical line perpendicular to the Point axis was drawn from this point. The intersection point on the Point axis represented the score under the determined value of the predictor. For example, when CD4 was 1200 cells/μL, the score was 0 point; when BMI was 12 kg/m^2^, the score was 63 points. By analogy, the score of each predictor could be determined, and summed up. Similarly, after the total score was calculated, a vertical line was drawn from the point of the patient’s total score on the Total Points axis to the axis of survival probability (such as three-year survival probability or five-year survival probability). The intersection point on the axis of survival probability represented the patient’s three-year or five-year survival probability.

## Discussion

Although the survival of PLHIV has been improved significantly with the promotion of free ART, a rapid and accurate prediction can benefit the personalized management of PLHIV and the allocation of medical resources [26].

For prognosis, due to a longitudinal temporal logic between predictors and outcome, the cohort study is used to analyze the data and fit a prognostic model. Randomized controlled clinical trials are considered as a prospective cohort study with more rigorous inclusion criteria, which therefore can be used to establish a prognostic model. However, it has limitations in extrapolation. Due to the population selection bias and information bias, retrospective cohort studies are not suitable for constructing a prognostic model, while nested case-control or case cohort studies are more economical and feasible for studies with rare outcomes or expensive predictive factor measurements. To decrease the influence of the limitation, we took into account the survival time when performed PSM. Based on this nested case-control study of an HIV/AIDS ART cohort in Nanjing, the relationship between routine laboratory indicators and the survival probability of PLHIV was evaluated. A prognostic model (including CD4, BMI and HB) with satisfactory discrimination and calibration was developed to predict the three-year and five-year survival of PLHIV receiving ART. Then the result of this prognostic model was shown in the form of a nomogram.

Nomogram is simple, direct and effective in predicting the prognosis of PLHIV [24]. In this study, the multivariable Cox proportional hazards regression model indicated that the five factors (Shingles, CD4, BMI, HB and TC) were associated with the HIV/AIDS-related survival time. To overcome the limitation of a single predictor and simplify the prediction procedure, three detected factors (CD4, BMI and HB) were combined to construct a prognostic model to predict the three-year and five-year survival of ART-treated PLHIV, which exhibited a high consistency.

WHO clinical stage had a close association with PLHIV survival [13] but was excluded in the nomogram. The main reason was that there was a strong relationship between WHO clinical stage and CD4 in the current study, which caused multicollinearity. In addition, the laboratory indicators (CD4) usually are more sensitive in predicting survival rate of PLHIV than the clinical indicators (WHO clinical stage). In recent years, many researchers have reported that some laboratory indicators are connected with the survival of PLHIV. In this study, CD4, BMI and HB were significantly correlated with the survival of PLHIV and showed good consistency with these published studies [10, 16, 21, 26].

An obesity paradox was seen in this predictive nomogram of PLHIV, and those with high BMI had a low risk of death. This may be due to the fact that the protective effect of BMI helps preserve the immune system response and slow the progression of HIV [31]. There is some evidence that a higher BMI is associated with more robust CD4 recovery in ART-treated patients [32]. Previous studies also suggested that the immune reconstitution on ART was often the highest among overweighted patients [33].

DCA is commonly applied to assess the efficacy of specific clinical prediction models [34]. In this study, DCA was used to assess the potential clinical benefits of nomogram, which revealed that nomogram was more effective and accurate than a single indicator in forecasting the survival of PLHIV. Prediction models are always less powerful in predicting outcomes during a long time. With more samples in the future, the performance of prediction models might be improved.

The present model has a limitation. It was established based on a few easily collected and low-cost predictors due to the underdeveloped technology in the past. However, as the economy and technology evolve, clinical prediction models that involve a larger number of data (big data) will be developed. Hopefully, more complex models and algorithms based on machine learning and artificial intelligence will provide more benefits to medical workers, PLHIV and medical decision makers.

## Supplementary Information

**Additional file 1: Supplementary data.** Raw data of HIV/AIDS patients used and analyzed in current study. Note: Variable names and values are described as follows. Infection route (1 = Homosexual transmission, 2 = Heterosexual transmission, 3 = Other transmission); Gender (1 = Male, 2 = Female); Marital status (1 = Unmarried, 2 = Married); Art = time of initiating antiretroviral therapy; TB = tuberculosis (0 = No, 1 = Yes); Continuous diarrhea (0 = No, 1 = Yes); Continuous or intermittent fever (0 = No, 1 = Yes); Shingles (0 = No, 1 = Yes); WHO clinical stage (1 = stage I or II, 2 = stage III, 3 = stage IV); ALT = alanine aminotransferase; AST = aspartate aminotransferase; BMI = body mass index; CD4 = CD4 cell count; WBC = white blood cell; PLT = blood platelet; HB = hemoglobin; CR = creatinine; TG = triglyceride; TC = total cholesterol; FBG = fasting blood glucose; TBIL = total bilirubin; HBV = Hepatitis B Virus (0 = Negative, 1 = Positive); HCV = Hepatitis C Virus (0 = Negative, 1 = Positive); Status = survival status at the last follow-up Status (0 = alive, 1 = dead); End = observation end point.

## Data Availability

The datasets used and/or analysed during the current study are available from the corresponding author on reasonable request.

## Abbreviations

CD4: CD4 cell count
WBC: White blood cell
PLT: Blood platelet
HB: Hemoglobin
CR: Creatinine
TG: Triglyceride
TC: Total cholesterol
FBG: Fasting blood glucose
AST: Aspartate aminotransferase
ALT: Alanine aminotransferase
TBIL: Total bilirubin
TB: Tuberculosis
BMI: Body mass index
CD8: CD8 cell count
VL: Viral load
PSM: Propensity-score matching
ART: Antiretroviral therapy
PLHIV: People living with HIV
C-Index: Concordance index
DCA: Decision curve analysis
